# Pressure. A Qualitative Analysis of the Perception of Concussion and Injury Risk in Retired Professional Rugby Players

**DOI:** 10.3390/jfmk6030078

**Published:** 2021-09-21

**Authors:** Ed Daly, Adam White, Alexander D. Blackett, Lisa Ryan

**Affiliations:** 1 School of Science & Computing, Galway-Mayo Institute of Technology, H91 T8NW Galway, Ireland; ed.daly@gmit.ie; 2 Faculty of Health and Life Sciences, Oxford Brookes University, Oxford OX3 0BP, UK; adamwhite@brookes.ac.uk; 3 School of Life Sciences and Education, Staffordshire University, Staffordshire ST4 2DE, UK; alexander.blackett@staffs.ac.uk

**Keywords:** concussion, professional rugby union, long term health, brain injury

## Abstract

This study interviewed retired professional rugby union players (≤10 years since retirement) to discuss their careers in the game of rugby union. The primary aim of the study was to document their understanding of concussion knowledge and the analogies they use to describe concussion. In addition, these interviews were used to determine any explicit and implicit pressures of playing professional rugby as described by ex-professional rugby players. Overall, 23 retired professional rugby players were interviewed. The participants had played the game of rugby union (n = 23) at elite professional standard. A semi-structured individual interview design was conducted with participants between June to August 2020. The research team reviewed the transcripts to identify the major themes from the interviews using a reflexive thematic analysis approach. Four major themes were identified: (1) medical and theoretical understanding of concussion, (2) descriptions of concussion and disassociated language, (3) personal concussion experience, and (4) peer influences on concussion within the sport. These were further divided into categories and subcategories. The interviews highlighted that players did not fully understand the ramifications of concussive injury and other injury risk, as it became normalised as part of their sport. This normalisation was supported by trivialising the seriousness of concussions and using dismissive language amongst themselves as players, or with coaching staff. As many of these ex-professional players are currently coaching rugby (48%), these interviews could assist coaches in treating concussion as a significant injury and not downplaying the seriousness of concussion in contact sports.

## 1. Introduction

Rugby union has been a professional sport (for males) since 1995. It is a collision sport that is highly physical in terms of impact with opposing players, resulting in frequent contact events and collisions throughout the course of a match. The amalgamation of very high physical demands, combined with regular impacts with opposing players, for example, tackling, scrummaging or mauls, means that injury is an inherent risk of the game. In a study examining 12-month match exposure in professional rugby union players, it was proposed that accumulated training workload and recent match exposure influence a player’s current injury risk [1]. This study demonstrated that players who had played a high (≥35) or a low (≤15) number of matches in the previous year were more susceptible to injury.

This results in players potentially enduring numerous injuries over the duration of a season in professional rugby (81 per 1000 h (95% CI 63–105), and 3 per 1000 h (95% CI 2–4) during training), and a similar level of injury exposure (ice hockey incidence rates of 79 per 1000 h and rugby league injury incidence rates of 68 per 1000 h) when compared to other collision sports [2]. As such, overall player welfare is the collective responsibility of coaches, team owners and individual players in professional rugby, as it can have a direct impact on players’ physical and mental health [3]. At professional levels, compared to rugby at amateur levels, injury risk is significantly higher in terms of physical injury and concussion risk exposure during contact events [4]. In conjunction with these factors, professional players often downplay or underreport concussion incidence and physical injury during their professional rugby careers [5]. Many ex-professional rugby players tend to normalise pain and accept that injury is an integral part of the sport. These beliefs can be reinforced by the environment and become part of the “habitus” of the players involved [6]. The habitus of rugby players includes a subconscious and conscious level of conformity to highly masculine norms and behaviours. These are the accepted behaviours and norms that they *should* display when they are part of a highly masculine environment [7]. By choosing to accept pain and injury as part of professional rugby, this can evolve into a “culture of risk” for the players involved [8]. By extension, this culture of risk can develop into an unquestioned acceptance of physical injury and concussion risk as a normal part of the sport [8,9].

The latent pressure to accept pain and injury risk in sport can become culturally engrained within an athlete’s mindset [10]. It has been suggested that many athletes accept pain and consider their ability to tolerate pain a particularly strong personal characteristic [11]. In a group culture, this pressure can be pervasive and become intrinsically linked to the character of the individuals involved and can be exacerbated by coaching or support staff. Under these circumstances, many athletes may consciously or subconsciously perceive that showing physical or mental weakness could tarnish the perception or “superhuman” concept of elite athletes [12]. In professional athletic environments, such as men’s professional rugby, a tolerance to injury can be perceived as a characteristic that substantiates their position within a group of fellow professional players. Once concussion and injury risk are accepted as part of professional rugby, a further extension of the culture of risk is to decide not to disclose concussion to medical or coaching staff within a club setting [13]. 

How can the culture of wanting to play while injured be explained in sport either at amateur or elite level? Research suggests that acceptance of pain can stem from elite athletes trivialising and/or ignoring pain; in these instances, athletes may prefer to self-medicate and disregard medical advice in order to compete [14]. Alternative research noted that rugby players chose to play while knowingly injured because of the “sport ethic” of rugby, where self-sacrifice was a widely established accepted behaviour in the sport, and part of the culture of risk associated with the sport [15]. Furthermore, it can be argued that the culture of risk evident in professional sport is not evident in other occupations [16]. In tandem with this, there is an acceptance that injury is routine and normal in sport [17] and is exacerbated within rugby union by a willingness to participate whilst hurt [18]. 

The connection between professional athletes and their peers increases pressure to play while injured. The social relations in playing groups generate internal and external pressure to play while injured regardless of the long-term consequences to players’ health [19]. The existence of this culture of risk is reinforced by acceptance that injury is part of the game and an aspect that players need to accept to be an elite athlete [8]. This study sought to investigate retired players’ understanding of concussion in conjunction with the explicit and implicit sources of pressure experienced in professional rugby union.

## 2. Methods

### 2.1. Study Design

A reflexive thematic analysis was utilised for this research study [20]. The primary goal was to examine retired professional rugby union players’ understanding of concussion and concussion experiences within the professional game of rugby union. A secondary goal was to examine the latent (implicit and explicit) pressures of being a professional rugby player with regard to concussion incidence and injury risk (n = 23). A semi-structured guide for the interviews was developed to offer an exploratory account of the players’ playing background and experience with concussion. 

### 2.2. Eligibility Criteria and Sampling

This study utilised a qualitative research approach, using semi-structured interviews and reflexive thematic analysis. An exponential non-discriminative snowball sampling method was utilised, as the first participants recruited to the study cohort provided other referrals [21]. All included participants were retired professional men’s rugby union players who had ended their playing careers within the ten years prior to the commencement of the study (retirement span 2011–2019). The players described their experiences of being involved in professional sport (professional rugby clubs and/or international rugby union representative level) from their subjective perspectives.

#### Participant Characteristics 

The participants associated with this study had all participated in professional rugby union (n = 23). The following nations were represented: Ireland (n = 17), England (n = 1), Scotland (n = 3) and Australia (n = 2). Of the full cohort of players (n = 23), 14 had represented their respective countries at full international test level rugby (61%). The average career span (years) for the cohort was 9.3 (SD 2.7) years in duration and the average age at time of retirement from professional rugby was 30.8 years (SD 2.9). 

### 2.3. Ethics & Procedure

Ethical approval for this study was received via the Research Sub Committee of Galway Mayo Institute of Technology (GMIT; RSC_AC_23062020). Pilot research was gathered from discussions within the research team and exploratory pilot trials of the interview format with potential participants. Data were collected during interviews ranging from twenty-five minutes to seventy minutes in duration with a preliminary conversation with each participant to outline the rationale for the study and how the study would proceed. Each participant was given a participant information sheet with a consent form attached.

### 2.4. Procedure & Data Collection

Before each one-to-one interview (interviewer (ED) & interviewee), verbal consent was attained, and the interview proceeded by using a standardized series of questions across all interviews. Participants were informed that all information gathered would be treated as confidential and anonymised for the purposes of this qualitative study. Post interview, all transcripts were read and verified by an independent reviewer (LR). The data from the interviews were collected sequentially from the participants and the transcripts were analysed once all interviews were completed. The main purpose of the study was to document the pressures of playing professional rugby union as described by a cohort of ex-professional rugby players. In addition, these interviews were used to ascertain their levels of concussion knowledge and the analogies they used to understand concussion. 

### 2.5. Data Analysis

After data collection from the interviews, each participant was given an opportunity to comment on the recording and offer clarifying comments on their personal interview. The audio recordings of the interviews were transcribed to MS Word format by the lead researcher (ED). After the transcription process, all transcripts were compared to the audio recordings of the interviews. Using this process for syntax correction, final amendments were made to the transcripts to generate the first draft of interview manuscripts (n = 23). Data were analysed thematically according to the Braun and Clarke reflexive thematic analysis approach, following an update to their original thematic analysis approach [20]. For this study, a critical realist framework was utilised to identify the players’ descriptions of concussion and their injury experiences in professional rugby [22]. From the 23 interview transcripts, the lead researcher identified eight subcategories, which were subsumed into five categories, resulting in four themes. Interview content and themes were independently assessed and reviewed by the third author to assist and finalise the thematic analysis process [23].

### 2.6. Researcher Background

The research team nominated ED as the primary interviewer to collect data. This was based on his previous experience as part of a professional rugby organisation and experience in gathering qualitative data. It was determined that the ex-professional players would be more responsive and/or more open in their interview responses with ED as compared to other members of the research team. This could be attributed to the presence of a male interviewer (ED) with prior experience of being part of a professional rugby organisation that would be conducting interviews with male participants [24,25]. This was viewed as a positive aspect with respect to the recruitment of participants for the study.

## 3. Results

This study examined the pressures associated with being a professional rugby player and the players’ understanding of concussion as an injury in professional rugby union. In this study, four major themes were identified by the authorial team. Based on significance, the lead author identified a hierarchical order for these four themes: (1) Medical and theoretical understanding of concussion; (2) Descriptions of symptoms and disassociated language; (3) Concussion experiences, misunderstanding of subconcussive impacts, categories of concussion; and (4) Peer influences on concussion within the sport. Table 1 illustrates the composition of these themes with regard to the categories and subcategories. Throughout the interviews and the process of theme identification, a connecting theme of language emerged across the four major themes. 

### 3.1. Theme 1—Medical & Theoretical Understanding of Concussion

#### 3.1.1. Awareness of the Physiology of Concussion

All players interviewed had a long-term association with the game of rugby. With this association, they had developed a profound connection with the game from an early age, e.g., “*I fell in love rugby around 9–10 years of age*”. This early exposure demonstrated that rugby is a collision sport and has inherent dangers, including concussion risk. The majority of players recalled being coached from an early age to tackle correctly in an attempt to mitigate concussion risk. These skills, when taught correctly, translated into appropriate tackle technique as a professional rugby player: “*I got exposed to good coaching as I was growing up, that’s why I love the game and I was lucky enough that I could make a living from it as well*”. The interviewees expressed opinions that direct blows to the head and neck had the highest probability of resulting in concussion while playing the game: “*When the impacts are coming like you know, the brain shakes in the head*”, and “one of mine was where I got chinned like it was wasn’t it wasn’t a big collision to the head. It was a hip to the chin, and it was almost like you’d see in a boxing match like a punch to the chin”, or more clearly expressed as “I got kicked in the head and then there was a loss of consciousness”.

This anecdotal evidence is supported by injury surveillance evidence, where the highest incidence of concussion occurs in the tackle and/or the contact areas of the game (i.e., tackles, rucks, mauls, etc.). This belief in improving tackle technique in order to reduce concussion risk in players is regarded as valid by many of the players, in terms such as, “*We can coach our young athletes in in their tackle technique as well, I think will be a would be a big defining factor*”. There was evidence to imply that incorrect technique leads to concussion and other physical injuries to fellow players: “*so many concussions probably around how you tackle and the breakdown area*”, and “*when you look back technically, he wasn’t, probably, you know, making tackles the way you should have been*”. Other players believed that certain strength and conditioning protocols could be implemented to reduce concussion risk: “*[i.e., increase the] amount of posterior work around the neck within their program as a prevention strategy*” *and* “*I think exercise, probably neck strengthening and I don’t know much about it, is possibly going to help (prevent concussion)*”. 

As the tackle and/or contact areas of the game are unlikely to change, some players were of the opinion that changes to current tackle laws will do little to mitigate against concussion risk. 

“Lowering the tackle height further, which has been discussed, would be a mistake. I think that’s going to lead to more concussions, because the reality is for someone who is 6′6”, if you got to defend two players, all of a sudden, you make a last second decision; my head is the thing that’s going to take the bang”.

#### 3.1.2. Non-Medical Descriptions & Understanding

While many of the players had a clear knowledge of the mechanism of concussion, the language used was frequently framed in non-medical terms, using analogies to describe the effects “*that kind of Deja vu feeling the kind … where the white stars kind of appear in the corner of your eyes*”. It was apparent that the injury was unclear and unnerving for players: 

“you got concussed, but it was a massive brain fog. It’s almost like when you wake up from a really good sleep and your alarm was going off. But you’re not quite sure exactly where you are at that moment”.

This sense of disorientation or uncertainty on a temporary basis had led the players to believe that concussion may not have been as serious as other injuries as they could still continue to play. 

“It’s a scary feeling when you’re in this situation …… in front of 50,000 people and I’ve got absolutely no idea where I am or what’s going on. You look around like holy fuck what’s going on here? I got this concussion”. 

Assigning non-medical descriptive language, for example, “head knock” or “head bang”, to concussion, may have had the effect of reducing the gravity of concussion in a professional environment. The combination of this type of language and a misunderstanding of concussions created an environment where players could continue to play while symptomatic or choosing to ignore that they had a concussion. 

“I had a couple of bangs to the head …… at the 2015 World Cup. I did one return to play where I’ve got head knock, think against South Africa. I managed to play the next week but apart from that I think I had another HIA (Head Injury Assessment) to do…but I’ve never been knocked unconscious at all in my career”.

Many players chose to ignore “minor” concussions and believed that only being knocked unconscious deemed them to be concussed, which was clearly expressed in the descriptive language for concussions in their careers. 

### 3.2. Theme 2—Descriptions of Symptoms and Disassociated Language 

#### Understating the Injury and Using Casual Terminology

This disassociation between the injury and the language used to describe it became the normal manner to describe concussion. This type of language seemed to trivialise the injury and cause a disconnection between declarative and procedural knowledge by the players: “*I would have knocks and seen stars … knocked out of whack*”. It led to players deeming that they needed to be “knocked out” in order to be diagnosed with concussion: “*I wasn’t knocked out or anything, but just a bit wobbly and had gone through the whole protocol*”. It is apparent that players judged their readiness to continue to participate on this metric of being conscious or unconscious and were intentionally dismissive of concussion by using casual language to rationalise the injury. Other descriptions were used where concussions became normalised to the player or were considered an accepted consequence of his occupation. For example, one player described this phenomenon as “*a run of the mill concussion …… one was six weeks long*”. This was considered as a standard description which was strongly connected to the previously noted habitus of the environment in which they operated as “tough” professional players [7].

### 3.3. Theme 3—Concussion Experiences, Misunderstanding of Subconcussive Impacts, Categories of Concussion 

#### 3.3.1. Knock Out Blows and Misunderstanding Subconcussive Impacts

The parameters of concussion definitions as described by the ex-players were predominantly related to whether they were knocked unconscious or whether they remained conscious and able to compete.

“I know it is a kind of grey area, you know, there’s a lot of times, you know, there’s a misunderstanding of what I call concussion actually is, I suppose the easiest way to kind of get this across is that I never had a situation where I was out cold where I was like fully unconscious”. 

During the interviews, this led to comments about subconcussive hits, and if they had experienced these types of impacts: “if I got a bang to the head, my memory was wiped for the next 3 or 4 min … I had no idea where you are, then and all of a sudden the fog starts to lift (and continued to play)”. It was noticeable that interviewees did not regard these repeated impacts as subconcussive hits; instead, the lack of clarity about what defined a concussion was a dominant collective comment. An example of such a view was seen when one participated stated:

“You (are) like Christ, what day is it? And it’s like that instant for a long period of time and it is a hard one to explain the consciousness of a concussion ‘cause sometimes you get knocked out cold, sometimes you’re kind of there with it, and sometimes it’s your memories aren’t that clearly there, so it’s obviously a difficult one to explain, but it’s just sort of a complete reset of your consciousness at the time. “

#### 3.3.2. Categories of Concussion

An extension of using casual terminology was a categorisation of concussion using euphemistic language, such as one participant’s explanation: “*A significant difference from that sparkly glitter stars (subconcussive impact) in your eyes to when you get a full-blown concussion. It’s important that you know what they feel like*”. The connection between a “full blown concussion” with unconsciousness and other concussion experiences where the player could continue was interesting to note. In medical terms, while assessing a concussion, medics can categorise concussion symptom severity using various assessment tools at pitch side or in a clinical setting. The players interviewed had their own stratification:

“I always sort of categorized into maybe 3 different areas; extreme is getting knocked out. Then not getting knocked out cold where you don’t know what’s happening, you’re conscious. Then the kind of where you got a big bang to the head, your vision kind of goes; stays with you for a while, it doesn’t go away, can’t really function all that well.”

What became explicit from some players was that they were unaware of the cumulative effects of concussion. These cumulative effects may have short- or long-term effects regardless of what category of concussion they had experienced: “*the small episodes are shaking your brain, you know, even some doctors seem to think that they can be as bad as the other ones as well*”. As concussion frequency escalated in some players, recovery times from concussion increased concurrently. 

“I had five (diagnosed) concussions in my career, but we both know the vast majority of players have a lot more than that, one thing that I always took a long time to recover from a concussion, even very minor ones, like I’m talking three weeks, maybe two months”.

#### 3.3.3. Unacknowledged or Hiding Concussion Symptomology 

Due to the latent pressure associated with a professional rugby environment, for example, contract and financial issues, it was a common trait for players to feel comfortable being dismissive of concussion. Players expressed this in terms of “*I remember multiple times during my career being very, very dazed on the field*” and in a more subtle sense of when they “*weren’t cognitively present during the course of the game*”. Many of these occurrences were a clear display of being in an overtly masculine environment and not willing to show any form of weakness as a professional rugby player. 

In tandem with these latent pressures, there was evidence to suggest that players are willing to compromise concussion test protocols in an attempt to remain in the game and hide their concussions, 

“I was just talking to this friend of mine who just retired last year. The protocols that were in place, we thought were fairly simple. And we thought that we could kind of trick them …… answer slower and even if you do have to go off and take the test, you’re going to pass”. 

Some players had other methods to strategize around the Sport Concussion Assessment Tool (SCAT) protocols: 

“I remember actively practicing those tests, trying to figure out the months of the year backwards, so I could do it or it’s ‘apple elbow carpet bubble saddle’. So even when I was concussed, I could go into autopilot and beat those tests … you can beat the system”.

Even when players felt that they did not play whilst concussed, their comments acknowledged that fellow players continued to play while symptomatic: “I’ve without doubt seen people that have been either hit badly or kind of a bit spaced”. 

### 3.4. Theme 4—Peer Influences on Concussion within the Sport

#### Sporting Culture, Reinforced Social Norms

When commenting on the rugby culture that infused their early sporting experiences, it was assumed that playing rugby carried an associated injury risk: “*Even that little bit of stars, that’s a concussion, I don’t know how many times I got that in my career even all the way up as a kid*”. This long-term chronic injury risk was reinforced by culture or familial influences: “*You know what you’re signing up for when you’re playing*”. The inclusive fraternal influence of parents, coaches and peers may not have been openly expressed but it was latently implied: 

“rugby was obviously a massive sport where I was from, my father was big into rugby, the school (I attended) was a big rugby school” and “I was told afterwards (experiencing a concussion) that I said, ‘please don’t take me off, my father kill me’ if you take me off’”.

To be perceived as physically weak and conceding to concussive injury was a source of humiliation for players. This was evidenced by comments such as: 

“it was pretty similar with the delayed (playing while symptomatic) concussions, just an incident but then afterwards you really didn’t feel good at all, I mean, it’s not something that you flag on the pitch”. 

The need to keep concussions hidden was deemed as a necessary action to protect their masculinity: “at the time and with an ego, I just tried to ignore it (concussion) as much that I could, so maybe I didn’t pay attention to it”. 

This manifested in a culture where players felt pressure from coaches and peers to be declared fit to play matches because “you’re not going to be the one to report yourself; then you’re out for the protocols, which could be up to two, three or four weeks”. Some players voiced opinions where they did not feel secure in declaring their symptoms to coaches and peers: “like this is the truth, it’s very hard to tell because in my early career I didn’t even report, I just got back to training and playing”. In other instances, players were unsure whether or not they were concussed, and chose to say nothing: “I didn’t tell anyone that I was concussed, so I played on, but I didn’t know either”. The undercurrent of pressure manifesting from peers and coaches was invariably directed back towards the players: 

“it is entirely reliant on the honesty of the players. As I said, the players are fully aware of it (pressure to play), they are competitive animals and they’re mad to play, particularly as well when you take in other variables like a guy coming out a contract or fighting for his position”. 

The risk was inherent during competitive matches and, interestingly, was an accepted part of preparation or training sessions leading up to gameday. From examples of concussion incidence discussed by the players, it was described as an accepted aspect of training. 

“I got knocked out by my flatmate at the time in a big contact (training) session, two egos colliding, and I ended up coming out the worst. I got knocked out cold. Sort of one of those ones where it’s in the highlights of training and people laughing at it because someone got knocked out cold”

If players received a blow to the head and were possibly symptomatic, it was understood that they would return to play: “(I) went off to get stitches and didn’t feel right …… then the one (concussion) after that which was in training that was just in a maul”, which was the widely accepted practice. 

With the benefit of hindsight after retiring from the game, many participants believed that a cultural shift is required in the game. Most participants were aware of concussion being discussed during their professional careers, but a significant emphasis may not have been placed on disclosing concussions or ongoing symptomology: “*In hindsight now, I should have just told the coaches whatever, I was just not right; yeah (taking) a week out, you know that distinction between a concussion and just having a very hard game, maybe talk a little bit more about*”. According to one participant, in order to implement meaningful change and avoid the current issues of hiding concussion symptoms or how concussion is discussed within the game, “*there needs to be a change and I think it needs to be cultural change; you stop accepting that this (hiding concussion symptoms or concussions) needs to be part of it …… and getting back on a field that is not necessary.”* It is evident that cultural shifts to manage pressure within the game cannot occur in isolation and need to be broad and *momentous,* “*whereas in years gone by, it was sort of seen as a badge of a badge of honour, where is now. I think boys are sort of saying ‘look, that’s not cool’. That’s an important thing. ‘cause obviously pressure from your teammates and that sort of environment*”.

## 4. Discussion

A key finding from this study was the language that male rugby players used when speaking about concussion and their descriptions of pressure in professional sport. The language associated with concussion was influenced by rugby culture and seemed to be quite dismissive of concussions or concussive symptoms. The language used highlighted the various sources of pressure that professional players experience as elite athletes. Many of the obstacles that players faced when attempting to reveal concussions were latent in nature. This unsaid or latent pressure was underpinned by the understanding that saying nothing was a preferable option for their careers. In a historical sense, the emergence of rugby culture is a legacy from the English public schools system that promoted sportsmanship and endurance with respect to pain tolerance [26]. This historic context partially explains the socialisation of rugby culture, which instilled a tolerance to pain that is generally at odds with what we find in broader society [9]. 

These pressures to accept pain were expressed and understood from an early age, which continued into their professional careers. Professional rugby is a very competitive environment, where the latent understanding is that players need to display durability, strength of character, physical strength, and a willingness to suffer in silence through physical pain and concussion. Many players did not fully understand the ramifications of concussive injury and other injury risk, as it became normalised as part of their sport [27]. In parallel, many players did not consider the long-term health implications of experiencing multiple concussions over the duration of a professional rugby career. This normalisation was supported by trivialising the seriousness of concussions either via dismissive language amongst themselves as players, or from coaching staff. The pervasive culture saw these risks as acceptable and legitimised the value of this ever-present risk for short-term success in the sport [11].

It was evident from the interviews that players experienced peer pressure from within the playing group. This pressure was intensified by fellow team members and coaching staff encouraging players to compete while injured. This practice may be understood as a “transfer” of the fear of failure from coaches to players as they (coaches) were under pressure to produce results and win games [8]. The latent pressure expressed by fellow players and coaches either directly or indirectly added to the overall burden that was experienced by the players. This was supported by the notion that being able to endure pain and play injured affirmed rugby players’ masculinity and affirmed their commitment to the club [19]. 

Similar comments were expressed by players who were not first choice or “fringe” players. Along with fringe players, there were players who were either recovering from injury or were injured near contract negotiations. These players were continuously uncertain of their futures as they felt excluded from the core playing group or that their “credit in the bank” would be easily forgotten, which led to doubts and issues with self-confidence. It is understandable that the manifestation of these various facets of pressure inevitably led to the players not disclosing concussive symptomology or choosing to continue to play while concussed. The participants in this study opted to dismiss long term physical and mental health primarily for financial gains and to maintain their personal status as professional rugby players. They managed to rationalise and trivialise the seriousness of concussion, either willingly, or unknowingly by a lack of knowledge about the consequences of multiple concussions and subconcussive impacts during their professional careers. Whether these participants used trivialising language to avoid disclosing concussions or did not understand the gravity of their concussive injuries warrants further investigation. The culture of risk expressed by the ex-professional players in this study clearly accepted that injury risk, including concussion, was an accepted part of the game. In this respect, the players accepted the associated implicit and explicit pressures because they wanted to succeed and be prepared for the stringent demands of elite rugby. It is evident from this study that medics and coaches cannot fully rely on players disclosing concussions or concussive symptoms.

In this regard, players used rationalising language to dismiss concussion or underreport concussion incidence. This was supported by self-imposed implicit pressure and explicit pressure applied by peers and coaches. These verbal pressures were expressed through direct and indirect language from peer groups or by coaches within the organisation. It could be argued that much of the language used was due to a lack of understanding of concussion and the mechanisms of being concussed or external pressures on coaching staff to produce results. In these instances, dismissive language or subtle language cues were used to normalise and rationalise concussion as an injury. This study, therefore, provides valuable detail to inform current professional, amateur and young players involved in collision sports. This findings from this study may also influence older retired players in a positive manner by highlighting areas of awareness and education for their long-term brain health. Due to the lack of research in this area, this study is the first of its kind to examine the pressures on professional rugby players, their understanding of concussion and the language they use to describe concussion. 

Many of the participants in this study were dismissive of concussion when they played professionally. Now that many of these ex-professional players are currently coaching rugby at amateur, elite, and professional levels (48%), it is interesting to note their current views on concussion. These interviews highlight the continuing need for education of coaching staff at all levels on the signs, symptoms, and recognition of concussion. These data could assist in eliminating outdated beliefs and the recycling of substandard practice associated with concussion and injury risk. These interviews provide valuable detail on the understanding, thoughts and language used by ex-professional players. This knowledge could assist coaches in understanding the importance of treating concussion as a significant injury and not downplaying the seriousness of concussion in contact sports.

### 4.1. Reflexivity

When examining the research process in this study, it is appropriate to acknowledge that the primary researcher attempted to remain critically aware throughout the process. It was notable that the lead researcher built rapport with the participants because of prior experience in professional rugby. Many of the participants offered forthright and authentic replies to the interview questions as a consequence. 

### 4.2. Study Limitations 

At the outset of this study, it was anticipated that interviews would be conducted with elite female rugby players; however, this did not materialise. Research into female elite players and their experiences of concussion is an area that warrants further research. This research paper included players who had retired in the previous ten years and may not be reflective of current practice. It is important to acknowledge that the research team involved in this paper have experience in professional rugby union and therefore the interpretation of the findings is through the lens of this previous experience.

## 5. Conclusions

There exists a disconnect between the language used by players and medical staff when discussing and reporting concussion incidence in the game of rugby. A thorough understanding of how players describe their symptoms is important to enhance recognition of concussion. Future research in this area would require a full discourse analysis of the language that is used by both male and female rugby players at amateur and elite levels of the game. As rugby union is a global sport involving multiple nations where different languages are spoken, discourse analysis can help identify whether these trivialisations of concussion and subconcussive symptoms occur throughout the professional world of rugby. This study also highlighted the fact that many players are dismissive of concussion/subconcussive hits and may not reveal their symptoms to coaches or may downplay their symptoms. Coaches and medical staff should therefore not include players in the decision-making process regarding return to play and should enforce concussion protocols.

## Figures and Tables

**Table 1:** Retired players understanding and descriptions of concussion in professional rugby.

Theme	Category	Sub-Category	Terminology	Selected Illustrative Comments from Players
Medical and theoretical understanding of concussion	Awareness of the physiology of concussion Non-medical descriptions	Mechanism of concussion Use of analogies to understand and describe symptoms	Impact, whiplash injury, shaking the brain, chinned Déjà vu, brain fog, deep sleep, being drunk, pressure in the head, disorientated, foggy, deep sleep, blacked out	“*get the impact there going one direction and the impact sends him another direction and it’s actually not them hitting the ground. It’s the movement of their head at that speed.*“ ***(P6)*** “*it’s a blinding concussion…… like a big charge, but it just went through my whole body and then boom, I fell on the ground*”*. **(P3)*** “*It was like being blackout drunk and not remember anything for hours later*”*. **(P5)*** “*You just feel, you know, your head full of pressure, I had that mild symptoms, a total pain in the back area or between your eyes and then just tension*”*. **(P21)***
Descriptions of symptoms and disassociated language	Understating the injury and using casual terminology	Dismissive; a non-serious injury	Crack, head knock, bang to the head, run of the mill concussion, Spots, stars, blurry vision, bell rung, dizziness, in a haze	“*that spark sort of thing is when you’re going to make that tackle and your head in the wrong spot. If someone really winged you in contact, that’s what I’m talking about with those one*”*. **(P16)*** “*To be honest, I’d say, you know, it was one of what do they call having your bell rung or something like that*”*. **(P18)***
Personal concussion experiences; misunderstanding of sub concussive impacts, categories of concussion	Knock out blows and sub concussive impacts	Knock out blows experience	Numbness, knocked out, panned out, out cold	“*I was knocked out to a point where it’s just a real numbness*”*. **(P3)*** “*I got knocked clean out and I just remember being brought in the car after I’ve been to the hospital*”*. **(P11)*** “*I was out cold in the field, tongue going down my throat*”*. **(P15)***
		Understanding; concussion and sub concussive impacts	Not fully knocked out, wobbly, gradual onset of symptoms, headaches	“*I know it is a kind of grey area, you know, there’s a lot of times, you know, there’s a misunderstanding of what I call concussion actually is, I suppose the easiest way to kind of get this across is that I never had a situation where I was out cold where I was like fully unconscious*”. ***(P17)*** “*I took a knock to the head and that was another gradual onset like initial thing was quite painful and the session was finished, very hot day as well. It was horrible because they’re terrible headache and you know I didn’t really want to talk to anyone or anything like that*”*. **(P22)***
		Categories of concussion	Minor concussions, strata of concussions, small episodes	“*It’s like a minor concussion and you play on, adrenaline gets through, but the next day your neck and your top your head would be sore to touch*”*. **(P10)*** “*The minor ones were, like, suppose any Sunday after a game it will be you’re sore everywhere. Like my neck and head would have been sore. And for the three or four days and it was like I could feel the side effects. Vision was slightly blurry, I wasn’t myself*”*. **(P10)***
		Unacknowledged or hiding concussion symptoms	Shake it off, get on with it, not being right, temporarily not cognitively present	“*You feel like you were there before or yesterday or a week before, and that would be a constant thing where you get knocked all different angles*”*. **(P10)***
Peer influences on concussion within the sport	Sporting culture, reinforced social norms	Influences on staying in the game	Loyalty, not admitting being injured, badge of honour, pressure from teammates	“*If you were able to stand up and play on and tackle the fellow in front of you and carried ball and everything, you’re not coming close enough. You’re not concussed enough to go off like, it kind of goes back to the point, that unless you’re asleep on the field and can’t actually stand up, you’re staying, and you play on, was the prevalent attitude*”*. **(P1)*** “*Players are pretty sharp these days as well as you know, it’s not just a case of ‘man up and get on with it’*”*. **(P2)*** “*The mentality of the whole sport around concussion, there was no talk about them*”*. **(P8)*** “*I understand guys put pressure on themselves to keep playing and toughen up and you know that is rugby for sure*” ***(P12)*** “*I think that sort of is encouraging, whereas in years gone by, it would be sort of seen as a badge of a badge of honour, whereas now, I think boys are sort of saying ‘look, that’s not cool’. That’s an important thing ‘cause obviously there’s pressure from your teammates in that sort of environment*”*. **(P12)***
